# Exploring staffing and hospital factors associated with WardPharm-1 filing in Japan: a nationwide observational study

**DOI:** 10.1186/s40780-026-00595-x

**Published:** 2026-06-19

**Authors:** Masayuki Kaneda, Maho Taguchi, Hiroaki Yamada, Akihiro Koide

**Affiliations:** 1 Laboratory of Regulatory Science, Yokohama University of Pharmacy, Yokohama, Japan; 2 Department of Pharmacy, Kawasaki Memorial Hospital, Kawasaki, Japan

**Keywords:** Hospital pharmacists, WardPharm-1 filing, Pharmacist-to-bed ratio, Workforce distribution, Team-based care, Healthcare system in Japan

## Abstract

**Background:**

The healthcare system of Japan faces mounting challenges, including population aging, medical complexity, and workforce shortages. Hospital pharmacists are expected to ensure the safe and effective use of medicines and contribute to multidisciplinary care; however, their availability and functions remain uneven. The “Ward Pharmaceutical Care Fee I” (WardPharm-1) serves as an indicator of the implementation of advanced ward-based pharmaceutical care. This study investigated the national trends and associated factors of WardPharm-1 filing, focusing on pharmacist-to-bed ratios and hospital characteristics, to explore factors that may enable high-quality pharmacist services.

**Methods:**

We examined national open datasets from the Ministry of Health, Labor, and Welfare for fiscal years 2021–2023, linking the “List of Registered Medical Care Providers (Medical)” and the “Hospital Bed Function Report.” Hospitals eligible for WardPharm-1 filing were identified, excluding psychiatric facilities and those not fulfilling minimum staffing standards. Per-100-bed staffing for eight healthcare professions and hospital bed counts were determined. Logistic regression and receiver operating characteristic (ROC) curve analyses determine the association between pharmacist staffing (Ph/100 beds) and WardPharm-1 filing, stratified by hospital type, Diagnosis Procedure Combination (DPC) group, and regional population density.

**Results:**

Among approximately 5,800 eligible hospitals annually, 31%–34% filed WardPharm-1. Pharmacists per 100 beds (Ph/100 beds) showed the strongest association with filing (odds ratio ≈ 1.2 yearly). Median Ph/100 beds were about 5.2 in filing hospitals and 2.6 in non-filers, with ROC cutoffs of 3.7–4.0 (AUC 0.83–0.84). Filing was most frequent in general hospitals (~ 50%) and DPC university or specified hospitals (~ 90%) and least frequent in long-term care hospitals (~ 3%) and depopulated regions (< 25%). Adjusted analyses confirmed that Ph/100 beds remained significantly associated with filing, though effect sizes were modest.

**Conclusions:**

Higher pharmacist-to-bed ratios were associated with higher WardPharm-1 filing rates. Filing was more common in larger and higher-function hospitals and less frequent in smaller or lower-density settings, patterns consistent with uneven workforce distribution. Although causality cannot be inferred, pharmacist staffing levels may serve as a practical indicator related to WardPharm-1 implementation.

**Supplementary Information:**

The online version contains supplementary material available at https://doi.org/10.1186/s40780-026-00595-x.

## Background

The healthcare delivery system of Japan is a critical foundation for safeguarding public health and ensuring that people can live with peace of mind [1]. Patient and community needs for healthcare have been increasing and diversifying; patient profiles continuously changing; and relationships with long-term care and daily life becoming more interwoven. Simultaneously, the environment surrounding healthcare is undergoing a rapid transformation due to population aging, advances in medical technology, tighter constraints on public finances, a shrinking labor force, and faster-than-expected progress in information and communications technology (ICT). Against this background, there exists an urgent need to establish an environment in which everyone can access healthcare without anxiety [1]. To this end, we must look ahead as far as possible and design a forward-looking, constructive pathway for functioning of healthcare delivery and healthcare professionals in the future [2].

For sustainable maintenance of Japan’s healthcare sector, it is essential to improve the productivity and added value of healthcare professionals; the expertise and insights of pharmacists are particularly critical contributors to this goal. Building on the professional knowledge developed through 6-year pharmacy education, pharmacists must assume efficient, high-productivity roles that can address workforce shortages [2]. Specifically, it is necessary to reform the work structure centered on dispensing to strengthen functions that are core to the profession—such as analyzing prescriptions, advising patients and other professionals, and developing pharmacotherapy protocols. Through these efforts, pharmacists proactively demonstrate their presence as members of multidisciplinary teams, acting as professionals in pharmaceutical services [2].

In hospitals, team-based care has advanced through ward assignment of pharmacists and a collaboration with other professions. Beyond managing patients’ own medications and medication adherence on the ward, pharmacists are expected to further enhance the effectiveness and safety of pharmacotherapy by proposing treatment plans to physicians—including monitoring therapeutic effects and adverse reactions and ordering tests as required [2]. Implementing these activities alleviates the burden on physicians and nurses [3–5] and has been widely reported to improve the quality of drug therapy and safe use of medicines across a broad range of settings such as acute care wards, subacute wards, chronic care wards, operating rooms, and oncology chemotherapy units [6–13].

Nevertheless, in the “Survey on Securing Hospital Pharmacists” conducted by the Japan Hospital Association in 2022 (2467 hospitals surveyed; 706 responses), 74.9% of responding hospitals reported lack of hospital pharmacists. Reasons cited for determining the required number of pharmacists included improving the quality of pharmaceutical services (90.5%), responding to requests from other departments such as clinical and nursing divisions (80.5%), and improving work schedules (71.1%) [14]. In the Ministry of Health, Labor, and Welfare (MHLW) project report on measures to secure pharmacists (questionnaire to 3183 hospitals; 631 valid responses), 64.8% of hospitals and 41.2% of community pharmacies recognized pharmacist shortages. Hospitals with pharmacist shortages reported impacts on routine work such as ward services (72.1%), overtime work (53.3%), and participation in team-based care (52.3%) [15]. Furthermore, the proportion of hospitals filing claims for the “Ward Pharmaceutical Care Fee I” (hereafter, WardPharm-1)—an indicator of clinical pharmacist activity—was reported as 35% in the 2022 Task Shift/Share Promotion Project report on the working conditions of hospital pharmacists (8194 hospitals surveyed; 2829 responses) [16] and 47% in a special survey on the results of the 2020 fee schedule revision (1500 hospitals surveyed; 494 responses) [17], i.e., less than half in both reports. These surveys suggest that hospital pharmacist services are not being completely implemented.

An MHLW Pharmaceutical and Food Safety Bureau study group on the training and quality improvement of pharmacists predicted a future overall surplus of pharmacists and also noted maldistribution across practice settings and regions. Securing hospital pharmacists was identified as an emergent issue, and attempts to address maldistribution—tailored to local circumstances and integrated with workforce measures in regional healthcare plans—were recommended. After reports and deliberations in the Social Security Council’s Medical Care Subcommittee, pharmacist maldistribution countermeasures were incorporated into the 8th Medical Care Plan [1, 18].

Despite the high expectations for hospital pharmacists, it is difficult to state that their expected functions are being completely verified under current conditions. This study used data from the “List of Registered Medical Care Providers (Medical)” and the Hospital Bed Function Report to examine differences between hospitals with and without WardPharm-1 filing. WardPharm-1 filing was used as a claims-based proxy indicator of ward-based pharmacist service implementation, with the aim of exploring factors that may facilitate the thriving of hospital pharmacists.

## Methods

### Databases

We used two open datasets viz., the “List of Registered Medical Care Providers (Medical)” and the Hospital Bed Function Report. The former is an open dataset published monthly by the Regional Bureaus of Health and Welfare (Hokkaido, Tohoku, Kanto-Shinetsu, Tokai-Hokuriku, Kinki, Chugoku-Shikoku, Shikoku, and Kyushu) on the acceptance status of notifications for medical fee items at hospitals and clinics that provide insured medical services [19]. The latter is an annual open dataset (aggregated for July each year) published by the MHLW and contains information on bed functions and staffing at medical institutions with general and long-term care beds [20].

We examined the data for 2021, 2022, and 2023. To align with the Hospital Bed Function Report month, we used the July files of the “List of Registered Medical Care Providers (Medical)” for each year. The number of hospitals registered in the July “List” was 8211 (2021), 8161 (2022), and 8133 (2023), and the number of hospitals published in the Hospital Bed Function Report was 7018, 6959, and 6974, respectively. We linked the two datasets using facility codes and hospital names. Because the Bed Function Report excludes psychiatric beds, we removed hospitals with ≥ 80% psychiatric beds (resulting in 6875 6806 and 6837 hospitals, respectively). We further excluded hospitals that did not fulfill legally required minimum staffing levels for physicians or pharmacists—judged to undermine the credibility of notifications—resulting in 6386 6064 and 5966 hospitals, respectively. Among these, hospitals billing any of the following were considered eligible to file WardPharm-1 and comprised the analytic sample: basic hospitalization fees for general wards, long-term care wards, tuberculosis wards, psychiatric wards, advanced treatment hospitals, or specialty hospitals. The resulting number of hospitals was 5833 (71.0%), 5512 (67.5%), and 5401 (66.0%), respectively. Population data were obtained from the 2020 Population Census basic tabulations, and habitable land area for municipalities was recorded from “Statistical Observations of Municipalities 2022” [21].

### Analytic items

As a claims-based indicator of high-quality hospital pharmacist services, we focused on WardPharm-1. Requirements to file WardPharm-1 include assigning a dedicated pharmacist to each ward and providing ≥ 20 h/week of ward pharmacist services. Required activities are (a) obtaining medication and injection histories and adverse event information from patients/families and identifying fundamental issues; (b) collecting and disseminating to staff the latest safety communications (e.g., emergency safety information, PMDA safety information, risk management plans, and recalls); (c) at admission, reconciling brought-in medications (names, strengths, dosage forms, etc.) and proposing a written medication plan to physicians; (d) checking for drug–drug interactions before the coadministration of two or more agents; (e) providing preadministration explanations to patients/families concerning medicines requiring special safety management; (f) performing necessary calculations (e.g., infusion rates and doses) before administration for such high-risk medicines; and (g) in addition to (a)–(f), performing the following as applicable: (1) under pre-agreed protocols, changing drug selection, dose, route, duration, or ordering tests; (2) actively proposing prescriptions to physicians regarding drug selection, dose, route, and duration; (3) proposing changes based on therapeutic drug monitoring and adverse event surveillance; and (4) performing appropriate aseptic preparation of anticancer agents [22]. It has been suggested that performing these WardPharm-1–required activities contributes to improved quality of pharmacotherapy and patient safety [23]. Therefore, we considered WardPharm-1 filing as an indicator expressing that hospital pharmacists are delivering adequate pharmaceutical care.

Previous literature suggests a relationship between WardPharm-1 filing and the number of employed pharmacists, e.g., “increased staffing to file WardPharm-1” (all 34 national Rousai hospitals responding) [23]; “insufficient pharmacist staffing most commonly cited reason for not filing WardPharm-1” (Gifu Prefecture; 47/91 responding hospitals) [24]; and “across all hospital functions, hospitals wished to engage in ward pharmacist services if staffing were sufficient” (Kyushu, Yamaguchi, Okinawa; 262/896 responses) [25]. Therefore, we hypothesized an association between WardPharm-1 filing and the number of employed pharmacists. Because hospitals with more pharmacists may also employ more of other professionals, we compared staffing for physicians, nurses, assistant nurses, nursing aides, physical therapists, occupational therapists, and speech–language–hearing therapists—professions commonly working on wards. Using the total number of beds and staff counts of each hospital, we calculated per-100-bed staffing for each profession (e.g., pharmacists per 100 beds = Ph/100 beds) to adjust for bed scale. The full-time equivalent number of part-time staff in the bed function report is the number of working hours per week of part-time staff divided by the scheduled working hours per week of the facility. Bed counts (total, and by general, long-term care, psychiatric, infectious disease, and tuberculosis) were collected from the “List of Registered Medical Care Providers (Medical).”

To explore the relationship between hospital function and WardPharm-1 filing, we used Diagnosis Procedure Combination (DPC) group categories—university hospital main group, specified hospital group, standard hospital group, and non-DPC hospitals—available in the Bed Function Report. In DPC hospitals, hospital roles are evaluated based on factors such as the basic coefficient and function evaluation coefficients I (e.g., add-ons for infection prevention, medical safety, and WardPharm-1) and II (e.g., emergency care, complexity, coverage, and regionality).

We defined hospitals filing WardPharm-1 as “WardPharm-1 hospitals” and those not filing as “non-filers.” Hospitals that transitioned from non-filer in year t to filer in year t + 1 were defined as “new filers,” those maintaining filing for 2 or 3 consecutive years were defined as “sustained filers,” and those transitioning from filer to non-filer were defined as “withdrawn filers.”

### Regional classification of secondary medical areas

Japan had 335 secondary medical areas (SMAs) during 2021–2023. We classified the SMAs into three types based on the population distribution as follows: metropolitan (population ≥ 1,000,000 or population density ≥ 2000 persons/km), provincial city (population ≥ 200,000 or population 100,000–200,000 with density ≥ 200 persons/km), and depopulated (all others).

### Hospital type

Hospitals were categorized according to the composition ratio of general, long-term care, and psychiatric beds among total authorized beds as follows: “general hospitals” (general beds ≥ 80%), “long-term care hospitals” (long-term care beds ≥ 80%), and “care-mix hospitals” (neither of the above).

### Statistical analysis

Logistic regression analysis was conducted using the number of staff per 100 beds for each healthcare profession as explanatory variables and the presence or absence of WardPharm-1 filing as the dependent variable. When analyzing staff numbers per 100 beds, personnel were categorized in increments of 0.5 persons per 100 beds. To further examine independent associations, a multivariable logistic regression analysis was additionally performed including pharmacists per 100 beds (Ph/100 beds), number of beds, hospital regional classification, hospital type, and DPC functional category as explanatory variables, with WardPharm-1 filing status as the dependent variable. Adjusted odds ratios (ORs) with 95% confidence intervals (CIs) were calculated for each variable. Logistic regression and receiver operating characteristic (ROC) analyses were performed to calculate the cutoff (CO) values and the area under the ROC curve (AUC) for the number of pharmacists per 100 beds (Ph/100 beds) and the number of hospital beds required for WardPharm-1 filing. All statistical analyses were conducted using JMP^®^ version 18 (SAS Institute Japan Inc., Tokyo, Japan).

## Results

### Multivariable analysis of per-100-bed staffing, bed count, hospital function, and hospital type in relation to WardPharm-1 filing

Among the eight professions examined in this study (physicians, pharmacists, nurses, assistant nurses, nursing aides, physical therapists, occupational therapists, and speech–language–hearing therapists), the logistic regression revealed that the odds ratio (OR) for Ph/100 beds contributing to WardPharm-1 filing was ~ 1.2 each year and was the highest among professions (Tables 1A–C). The ORs for other professions ranged from 0.95 to 1.01, suggesting limited contribution to filing. Among the eight counted professions in surveyed hospitals, Ph/100 beds exerted the strongest positive effect on filing. In multivariate analysis of other factors, Ph/100 beds was associated with application of WardPharm-1 (OR 1.16, 95% CI 1.14–1.18, *p* < 0.0001). The number of beds and hospital structural factors (regional classification, hospital type, DPC functional classification, etc.) were also significantly associated with application status. These findings were consistent across all study years(Tables 2A–C).

### National WardPharm-1 filing status and relationships with Ph/100 beds and bed count

Nationally, the numbers (and proportions) of hospitals eligible to file WardPharm-1 were 5833 (91.3%), 5512 (90.9%), and 5401 (90.5%) in 2021–2023, with 1820 (31.2%), 1801 (32.7%), and 1856 (34.4%) actually filing, respectively (Table 3A). Median (IQR) Ph/100 beds among eligible hospitals were 3.26 (2.10–5.00), 3.33 (2.14–5.00), and 3.41 (2.17–5.15). Among WardPharm-1 hospitals, the median Ph/100 beds were 5.24 (3.92–6.71), 5.21 (3.92–6.80), and 5.24 (3.92–6.96) and among non-filers, the bed counts were 2.58 (1.83–3.75), 2.63 (1.85–3.76), and 2.63 (1.86–3.85), respectively (Table 3B). Median differences between filers and non-filers were 2.76, 2.58, and 2.61 pharmacists/100 beds, respectively. The Ph/100-bed CO values (AUCs) for being a WardPharm-1 hospital were 3.72 (0.84), 3.95 (0.83), and 3.73 (0.83), respectively (Table 3B). Median bed counts of WardPharm-1 hospitals were ≥ 2.1 times those of non-filers each year (ratios 2.19, 2.16, and 2.12, respectively).

### Three-year filing/withdrawal patterns and trends in Ph/100 beds and bed counts

Trends in Ph/100 beds and bed counts according to filing patterns are summarized in Supplementary Table S1. Sustained filers consistently showed higher Ph/100 beds than withdrawn or non-filing hospitals, whereas withdrawn filers demonstrated decreasing trends and new filers showed increasing trends. Median bed counts remained relatively stable across years but were generally larger in hospitals that sustained filing.

### WardPharm-1 filing according to SMA density type: Ph/100 beds and bed counts

According to the SMA category, WardPharm-1 filing proportions were highest in metropolitan SMAs and increased annually as follows: 42.3%, 43.8%, and 46.1% (all > 40%), respectively. Provincial city SMAs were 26.2%, 28.0%, and 29.2% (< 30%), and those in depopulated SMAs were 21.3%, 22.1%, and 23.1% (< 25%), respectively (Table 4). For Ph/100 beds, median counts and CO values increased with population density. The national CO values (AUCs) of 3.72 (0.84), 3.95 (0.83), and 3.73 (0.83) were lower than the metropolitan median values but higher than provincial and depopulated median values. Median bed counts were also higher in denser SMAs; in any SMA type, WardPharm-1 hospitals had approximately double the median beds of non-filers.

**Table 1:** Comparison of the contribution of each medical profession (per 100 Beds) to ward pharmaceutical care fee I filing

A					
Year 2021	Odds ratio	Lower 95% CI	Upper 95% CI	Estimate	*p*-value (Prob > Chi-Square)
Number of pharmacist	1.1965	1.1705	1.2235	0.1794	<0.0001
Number of nurses	1.0081	1.0063	1.0100	0.0081	<0.0001
Number of physical therapists	1.0080	1.0025	1.0136	0.0079	0.0047
Number of occupational therapists	1.0041	0.9903	1.0179	0.0041	0.5615
Number of physicians	1.0029	0.9982	1.0078	0.0029	0.2274
Number of speech-language-hearing therapists	0.9920	0.9655	1.0193	-0.0081	0.5602
Number of nursing aides	0.9881	0.9837	0.9924	-0.0120	<0.0001
Number of assistant nurses	0.9511	0.9447	0.9575	-0.0501	<0.0001

**B**					
**Year 2022**	**Odds ratio**	**Lower 95% CI**	**Upper 95% CI**	**Estimate**	**p-value (Prob > Chi-Square)**
Number of pharmacist	1.2037	1.1767	1.2318	0.1854	<0.0001
Number of physical therapists	1.0108	1.0050	1.0166	0.0107	0.0002
Number of nurses	1.0090	1.0071	1.0110	0.0090	<0.0001
Number of speech-language-hearing therapists	1.0077	0.9804	1.0361	0.0077	0.5865
Number of physicians	1.0013	0.9968	1.0061	0.0013	0.5704
Number of occupational therapists	0.9950	0.9806	1.0086	-0.0051	0.4847
Number of nursing aides	0.9855	0.9807	0.9901	-0.0147	<0.0001
Number of assistant nurses	0.9530	0.9459	0.9600	-0.0481	<0.0001

**C**					
**Year 2023**	**Odds ratio**	**Lower 95% CI**	**Upper 95% CI**	**Estimate**	**p-value (Prob > Chi-Square)**
Number of pharmacist	1.1952	1.1697	1.2219	0.1783	<0.0001
Number of occupational therapists	1.0093	0.9954	1.0232	0.0092	0.1878
Number of nurses	1.0081	1.0063	1.0100	0.0081	<0.0001
Number of physical therapists	1.0069	1.0013	1.0124	0.0068	0.0148
Number of physicians	1.0043	0.9997	1.0090	0.0043	0.0692
Number of speech-language-hearing therapists	0.9918	0.9653	1.0189	-0.0082	0.5515
Number of nursing aides	0.9842	0.9797	0.9885	-0.0160	<0.0001
Number of assistant nurses	0.9503	0.9430	0.9575	-0.0510	<0.0001

OR = Odds Ratio; CI = Confidence Interval; Ph/100 beds = pharmacists per 100 beds

**Table 2:** Multivariate logistic regression analysis of factors associated with WardPharm-1 applications, including number of beds, hospital function, and regional classification

A					
Year 2021	Odds ratio	Lower 95% CI	Upper 95% CI	Estimate	*p*-value (Prob > Chi-Square)
Number of pharmacist	1.1623	1.1410	1.1844	0.1504	<0.0001
Number of beds	1.0081	1.0063	1.0100	0.0031	<0.0001
Hospital Regional Classification	1.0080	1.0025	1.0136	-0.3034	<0.0001
Hospital Type	1.0041	0.9903	1.0179	-0.7823	<0.0001
DPC Functional Category	1.0029	0.9982	1.0078	-0.9710	<0.0001

**B**					
**Year2022**	**Odds ratio**	**Lower 95% CI**	**Upper 95% CI**	**Estimate**	**p-value (Prob > Chi-Square)**
Number of pharmacist	1.1670	1.1447	1.1903	0.1544	<0.0001
Number of beds	1.0030	1.0023	1.0036	0.0030	<0.0001
Hospital Regional Classification	0.7285	0.6505	0.8153	-0.3168	<0.0001
Hospital Type	0.4231	0.3703	0.4822	-0.8601	<0.0001
DPC Functional Category	0.3661	0.3047	0.4395	-1.0049	<0.0001

**C**					
**Year2023**	**Odds ratio**	**Lower 95% CI**	**Upper 95% CI**	**Estimate**	**p-value (Prob > Chi-Square)**
Number of pharmacist	1.1652	1.1439	1.1873	0.1529	<0.0001
Number of beds	1.0029	1.0023	1.0036	0.0029	<0.0001
Hospital Regional Classification	0.7257	0.6490	0.8109	-0.3206	<0.0001
Hospital Type	0.4285	0.3758	0.4871	-0.8476	<0.0001
DPC Functional Category	0.3878	0.3228	0.4657	-0.9472	<0.0001

OR = Odds Ratio; CI = Confidence Interval; Ph/100 beds = pharmacists per 100 beds

**Table 3:** Relationship between ward pharmaceutical care fee I filing status and Ph/100 beds and number of beds nationwide

A									
				Year2021		Year2022		Year2023	
Total number of hospitals				6386		6064		5966	
Number of hospitals eligible to file WardPharm-1				5833		5512		5401	
Proportion of hospitals eligible to file WardPharm-1(%)				91.3		91.0		90.5	
Number of hospitals filing WardPharm-1				1820		1801		1856	
Proportion of WardPharm-1 filing hospitals(%)				31.2		32.7		34.4	

**B**									
	**Year2021**			**Year2022**			**Year2023**		
	**Number of hospitals**	**Pharmacists per 100 beds (persons)**	**Number of beds (beds)**	**Number of hospitals**	**Pharmacists per 100 beds (persons)**	**Number of beds (beds)**	**Number of hospitals**	**Pharmacists per 100 beds (persons)**	**Number of beds (beds)**
All WardPharm-1-eligible hospitals	5,833	3.26(2.10-5.00)	126(70-224)	5,512	3.33(2.14-5.00)	130(72.3-233)	5,401	3.41(2.17-5.15)	135(76-240)
WardPharm-1 hospitals	1,820	5.24(3.92-6.71)	219(122-398)	1,801	5.21(3.92-6.80)	220(125-395)	1,856	5.24(3.92-6.96)	220(126-397)
Cutoff value (Area Under the Curve)		3.72(0.84)	181(0.75)		3.95(0.83)	183(0.75)		3.73(0.83)	175(0.75)
Non-filing hospitals	4,013	2.58(1.83-3.75)	100(60-175)	3,711	2.63(1.85-3.76)	102(60-177)	3,545	2.63(1.86-3.85)	104(60-179)

Ph/100 beds and the number of beds (excluding cutoff values [area under the curve]) are shown as median (interquartile range)

AUC: Area Under the ROC Curve; WardPharm-1: Ward Pharmaceutical Care Fee I

There is no corresponding data in the cells with diagonal lines

**Table 4:** Comparison by hospital regional classification: ward pharmaceutical care fee I filing, pharmacists per 100 beds, and bed scale (2021–2023)

		Year 2021				Year 2022				Year 2023			
		Number of hospitals	Filing rate(%)	Pharmacists per 100 beds (persons)	Number of beds (beds)	Number of hospitals	Filing rate(%)	Pharmacists per 100 beds (persons)	Number of beds (beds)	Number of hospitals	Filing rate(%)	Pharmacists per 100 beds (persons)	Number of beds (beds)
Metropolitan-type hospitals	WardPharm-1 hospitals	872	42.3	5.73(4.44-7.31)	218.5(117-400)	844	43.8	5.74(4.42-7.36)	216(117.25-392.75)	886	46.1	5.80(4.34-7.60)	221.5(120.75-400)
	Cutoff value (Area Under the Curve)			4.24(0.82)	203(0.74)			4.23(0.82)	182(0.73)			4.31(0.81)	182(0.73)
	Non-filing hospitals	1,191		2.97(2.02-4.17)	107(60-175)	1,084		2.95(2.00-4.17)	108(60-177)	1,038		3.04(2.08-4.27)	110(60-180)
	All metropolitan-type hospitals	2,063		4.03(2.56-6.03)	141(80-261)	1,928		4.05(2.55-6.06)	143.5(82-270)	1,924		4.17(2.67-6.26)	150(88-287)
Provincial city	WardPharm-1 hospitals	775	26.2	5.05(3.85-6.31)	240(145-404)	785	28.0	5.00(3.81-6.36)	238(142-405.5)	795	29.2	5.03(3.83-6.54)	230(137-401)
	Cutoff value (Area Under the Curve)			3.67(0.83)	175(0.77)			3.44(0.83)	175(0.77)			3.56(0.83)	175(0.77)
	Non-filing hospitals	2,181		2.5(1.79-3.64)	100(60-177)	2,019		2.60(1.85-3.70)	101(60-178)	1,924		2.54(1.82-3.77)	101(60-177)
	All provincial city-type hospitals	2,956		3.03(2.00-4.64)	122(67-220)	2,804		3.16(2.06-4.70)	125(70-229.75)	2,719		3.22(2.04-4.81)	128(71-230)
Depopulated	WardPharm-1 hospitals	173	21.3	4.00(3.08-5.01)	193(99-315.5)	172	22.1	3.98(3.13-5.01)	196(105-315.75)	175	23.1	4.02(3.14-5.11)	198(109-315)
	Cutoff value (Area Under the Curve)			3.01(0.81)	174(0.71)			3.01(0.81)	121(0.72)			3.01(0.81)	175(0.71)
	Non-filing hospitals	641		2.32(1.72-3.27)	99(58-170)	608		2.36(1.67-3.33)	100(60-255)	583		2.38(1.67-3.33)	100(60-173)
	All depopulated-area-type hospitals	814		2.63(1.86-3.68)	109(60-198)	780		2.68(1.85-3.78)	110(60.5-199)	758		2.78(1.91-3.93)	116.5(62.75-199)
All hospitals	WardPharm-1 hospitals	1,820	31.2	5.24(3.92-6.71)	219(122-398)	1,801	32.7	5.21(3.92-6.80)	220(125-395)	1,856	34.4	5.24(3.92-6.96)	220(126-397)
	Cutoff value (Area Under the Curve)			3.72(0.84)	181(0.75)			3.95(0.83)	183(0.75)			3.73(0.83)	175(0.75)
	Non-filing hospitals	4,013		2.58(1.83-3.75)	100(60-175)	3,711		2.63(1.85-3.76)	102(60-177)	3,545		2.63(1.86-3.85)	104(60-179)
	All hospitals	5,833		3.26(2.10-5.00)	126(70-224)	5,512		3.33(2.14-5.00)	130(72.3-233)	5,401		3.41(2.17-5.15)	135(76-240)

Ph/100 beds and the number of beds (excluding cutoff values [area under the curve]) are shown as median (interquartile range)

AUC: Area Under the ROC Curve; WardPharm-1: Ward Pharmaceutical Care Fee I

There is no corresponding data in the cells with diagonal lines

### WardPharm-1 filing hospital type: Ph/100 beds and bed counts

Filing proportions differed by hospital type (Supplementary Table S2). General hospitals showed higher filing rates and higher Ph/100 beds than care-mix and long-term care hospitals. In contrast, median Ph/100 beds in care-mix and long-term care hospitals remained below the corresponding cutoff values. Bed counts were substantially larger in filing general hospitals than in non-filers, whereas differences were modest in other hospital types.

### WardPharm-1 filing according to the DPC group: Ph/100 beds and bed counts

Across DPC categories (Table 5), the median Ph/100 beds among WardPharm-1 hospitals consistently ranked university main > specified > standard > non-DPC, indicating higher Ph/100 beds with higher hospital function. Regarding CO values, the values for specified hospitals slightly exceeded those of university main in 2021 and 2023, but as their functional levels are comparable, with higher hospital function generally corresponding to higher Ph/100-bed CO values. Among non-DPC hospitals, median Ph/100 beds for filers were lower than the CO values for university main and specified DPC hospitals and even lower than the median values of non-filers in those higher DPC groups; in 2021–2022, they were also less than the CO values for DPC standard hospitals.

**Table 5:** Comparison by DPC Functional Category: Ward Pharmaceutical Care Fee I Filing Rate, Pharmacists per 100 Beds, and Number of Beds (2021–2023)

		Year 2021				Year 2022				Year 2023			
		Number of hospitals	Filing rate(%)	Pharmacists per 100 beds (persons)	Number of beds (beds)	Number of hospitals	Filing rate(%)	Pharmacists per 100 beds (persons)	Number of beds (beds)	Number of hospitals	Filing rate(%)	Pharmacists per 100 beds (persons)	Number of beds (beds)
DPC university hospital main group	WardPharm-1 hospitals	76	93.8	7.68(6.76-8.44)	831(658.25-998)	76	95.0	7.64(6.91-8.71)	831(658.3-998)	75	94.9	7.74(6.98-9.11)	845(674-990)
	Cutoff value (Area Under the Curve)			4.81(0.82)	632(0.52)			6.86(0.94)	618(0.59)			4.89(0.76)	1160(0.50)
	Non-filing hospitals	5		4.13(3.14-7.44)	800(513-1157)	4		4.73(3.14-6.46)	707.5(615-1086.5)	4		4.47(2.95-8.56)	825(661.3-1099)
	All DPC university hospital main group	81		7.57(6.71-8.35)	830(648.5-1009)	80		7.50(6.84-8.60)	828.5(646.25-998)	79		7.73(6.96-9.11)	845(674-990)
DPC specified hospital group	WardPharm-1 hospitals	141	88.1	6.71(5.44-7.80)	578(480-685.5)	153	92.2	6.84(5.63-7.97)	565(457.5-676)	159	91.4	6.97(5.71-7.95)	570(470-680)
	Cutoff value (Area Under the Curve)			5.20(0.69)	473(0.53)			6.28(0.78)	852(0.46)			5.50(0.71)	628(0.59)
	Non-filing hospitals	19		5.16(3.94-6.70)	615(402-676)	13		4.96(4.23-5.97)	630(450.5-704.5)	15		5.43(4.92-7.00)	647(460-733)
	All DPC specified hospital group	160		6.58(5.25-7.73)	584.5(474-685)	166		6.59(5.51-7.90)	570(458.75-677)	174		6.92(5.60-7.94)	576.5(467.75-684)
DPC standard hospital group	WardPharm-1 hospitals	963	64.7	5.46(4.39-6.73)	300(195-399)	947	65.5	5.4(4.34-6.8)	296(191-388)	974	66.3	5.53(4.31-6.97)	292(195-393)
	Cutoff value (Area Under the Curve)			4.23(0.80)	285(0.62)			4.27(0.78)	285(0.61)			4.03(0.78)	272(0.61)
	Non-filing hospitals	526		3.44(2.58-4.54)	220(148-324.25)	498		3.57(2.63-4.52)	220(150-317)	494		3.64(2.51-4.73)	218.5(146.8-322)
	All DPC standard hospital group	1,489		4.81(3.52-6.20)	267(179-375.5)	1,445		4.76(3.54-6.23)	262(176.5-367.5)	1,468		4.88(3.60-6.35)	263(179-370.75)
non-DPC hospitals	WardPharm-1 hospitals	640	15.6	4.04(3.03-5.50)	110(72-167.75)	625	16.4	4.07(3.06-5.49)	112(73-169)	648	17.6	4.10(3.07-5.45)	115(72-171.8)
	Cutoff value (Area Under the Curve)			2.66(0.77)	83(0.58)			2.95(0.77)	93(0.58)			2.93(0.76)	93(0.57)
	Non-filing hospitals	3,463		2.47(1.75-3.51)	96(56-150)	3,196		2.5(1.78-3.56)	97(56-150)	3,032		2.5(1.79-3.61)	98(58-152)
	All non-DPC hospitals	4,103		2.67(1.86-3.93)	99(59-152)	3,821		2.75(1.90-3.95)	99(59-153)	3,680		2.78(1.94-4.00)	99(60-156)
All hospitals	WardPharm-1 hospitals	1,820	31.2	5.24(3.92-6.71)	219(122-398)	1,801	32.7	5.21(3.92-6.80)	220(125-395)	1,856	34.4	5.24(3.92-6.96)	220(126-397)
	Cutoff value (Area Under the Curve)			3.72(0.84)	181(0.75)			3.95(0.83)	183(0.75)			3.73(0.83)	175(0.75)
	Non-filing hospitals	4,013		2.58(1.83-3.75)	100(60-175)	3,711		2.63(1.85-3.76)	102(60-177)	3,545		2.63(1.86-3.85)	104(60-179)
	All hospitals	5,833		3.26(2.10-5.00)	126(70-224)	5,512		3.33(2.14-5.00)	130(72.3-233)	5,401		3.41(2.17-5.15)	135(76-240)

Ph/100 beds and the number of beds (excluding cutoff values [area under the curve]) are shown as median (interquartile range)

AUC: Area Under the ROC Curve; WardPharm-1: Ward Pharmaceutical Care Fee I

There is no corresponding data in the cells with diagonal lines

## Discussion

Nationally, WardPharm-1 filing increased over the study period; however, the overall filing rate remained limited. According to the Japan Hospital Pharmacists Association guideline “How to Promote Ward Pharmacist Services, Ver. 1.2,” ward pharmacist tasks are generally divided into services provided before administration and those provided after administration, and implementing both may contribute to high-quality practice [26]. In addition, the filing rate for the Pharmaceutical Management and Guidance Fee has been reported as 64.8% [27], suggesting that pharmacist services involving direct patient intervention are already widely implemented. Taken together, increasing the proportion of hospitals filing WardPharm-1 may be important.

Regarding each medical profession, the odds ratio (OR) for pharmacists per 100 beds (Ph/100 beds) in relation to WardPharm-1 filing was 1.2 (*p* < 0.0001), indicating a 20% higher likelihood of WardPharm-1 filing. Meanwhile, although the ORs for nurses and physical therapists (PTs) were approximately 1.01, the p-values were statistically significant across all study years and the lower bounds of the confidence intervals exceeded 1.0. Even after adjustment for hospital structural characteristics, Ph/100 beds showed an association with WardPharm-1 filing. Nevertheless, given the observational design, these associations should be interpreted cautiously and should not be construed as causal.

Nurse staffing levels are regulated according to hospital function, and hospitals with higher functional levels tend to employ more nurses. As shown in Results 5 and Results 6, hospital function appears to be associated with the WardPharm-1 filing rate; consequently, the observed OR between the number of nurses and WardPharm-1 filing may reflect this positive association. For PTs, exploratory analyses using the same dataset indicated that non-DPC and care-mix hospitals had higher numbers of PTs per 100 beds than hospitals in other functional categories, and that WardPharm-1 hospitals had more PTs than non-filing hospitals (data not shown). Hospitals with wards requiring larger numbers of PTs, such as convalescent rehabilitation wards, may have fewer wards eligible for WardPharm-1 filing, which could make WardPharm-1 filing more feasible.

These findings may also be interpreted from the perspective of multidisciplinary collaboration. Higher numbers of nurses and physical therapists may reflect broader institutional capacity for team-based care. Clinically, nurses and physical therapists can provide pharmacists with information on medication-related problems, patients’ functional status, and rehabilitation progress. Such information may complement pharmacists’ own pharmaceutical monitoring and may help support pharmacotherapy optimization and medication safety. This collaboration may facilitate the expansion of pharmacists’ professional functions in hospital settings.

An association between pharmacist staffing and WardPharm-1 filing has been reported previously [27]. In our study as well, hospitals with higher filing rates tended to have higher Ph/100 beds. However, hospitals with more pharmacists often employ larger numbers of other healthcare professionals, and other factors may also contribute to whether WardPharm-1 is filed; therefore, these findings should be interpreted cautiously.

Filing rates varied by hospital function, size, and regional population density, and tended to be higher in higher-function and larger hospitals, and lower in smaller hospitals and regions with lower population density. These results are consistent with nationwide data indicating shortages of hospital pharmacists in many secondary medical areas (SMAs) [28]. On the other hand, compared with DPC hospitals that provide a wide range of functions, non-DPC hospitals may be able to focus more on WardPharm-1-related activities and thus meet filing requirements with relatively fewer pharmacists. In addition, exploratory analyses using the same dataset suggested that, compared with areas of higher habitable-land population density, areas with lower density had fewer ambulance acceptances, lower maximum and minimum numbers of occupied beds, and potentially fewer patients per hospital; therefore, in lower-density areas, WardPharm-1 filing may be achievable even with relatively fewer pharmacists (data not shown).

However, several factors not included in the open datasets used in this study may have influenced our findings. For example, pharmacists’ years of experience and specialist qualifications may affect the ability and efficiency of implementing WardPharm-1. Pharmacists with specialist qualifications may not necessarily be directly involved in WardPharm-1 activities and may instead be assigned to specialized pharmaceutical services. Therefore, analyses that incorporate not only the number of pharmacists but also factors such as years of professional experience and engagement of specialist pharmacists in specialized duties may provide a more accurate understanding of the current situation.

In addition, even among hospitals with the same number of beds, differences in the number of admissions and discharges, bed occupancy rates, and average length of stay may substantially affect workload. These unmeasured factors may influence both staffing levels and the ability to implement WardPharm-1, potentially leading to residual confounding, and should therefore be considered a limitation of this study. Furthermore, hospital operational conditions during the COVID-19 pandemic may have temporarily altered staffing allocation and service delivery, which could have affected the observed associations. Therefore, caution is warranted when generalizing these findings beyond the study period and setting. Recent studies have reported efficiency gains through digital transformation (DX), task shifting, and interprofessional collaboration [29–40], and combining these initiatives with measures to address pharmacist maldistribution may be important for strengthening hospital pharmacy practice. Although these findings should be interpreted cautiously given the observational design, they may contribute to nationwide considerations regarding workforce policy and hospital pharmacy practice.

## Conclusion

This study explored factors associated with WardPharm-1 filing, which was used as a claims-based proxy indicator of ward-based pharmacist service implementation and one aspect of the professional functions of hospital pharmacists. Higher Ph/100 beds were consistently associated with WardPharm-1 filing, and hospital function, size, and regional characteristics were also associated with filing patterns. These findings suggest that pharmacist staffing and institutional context may be related to conditions that support ward-based pharmacist services and facilitate the professional functions of hospital pharmacists. Given the observational design, these results indicate associations rather than causal relationships.

## Supplementary Information

Below is the link to the electronic supplementary material.


Supplementary Material 1


## Data Availability

This study used only publicly available datasets from the Ministry of Health, Labour and Welfare (MHLW) and e-Stat. No proprietary or personally identifiable data were used. Additional data supporting the findings of this study are available from the corresponding author upon reasonable request.

## Abbreviations

WardPharm-1: Ward Pharmaceutical Care Fee I
DX: Digital transformation
DPC: Diagnosis Procedure Combination
ICT: Information and communications technology
